# *Porphyromonas gingivalis *induce apoptosis in human gingival epithelial cells through a gingipain-dependent mechanism

**DOI:** 10.1186/1471-2180-9-107

**Published:** 2009-05-27

**Authors:** Panagiota G Stathopoulou, Johnah C Galicia, Manjunatha R Benakanakere, Carlos A Garcia, Jan Potempa, Denis F Kinane

**Affiliations:** 1 Center for Oral Health and Systemic Disease, School of Dentistry, University of Louisville, Louisville, KY, USA; 2 Department of Biochemistry, University of Georgia, Athens, GA, USA

## Abstract

**Background:**

The oral pathogen *Porphyromonas gingivalis *has been shown to modulate apoptosis in different cell types, but its effect on epithelial cells remains unclear.

**Results:**

We demonstrate that primary human gingival epithelial cells (HGECs) challenged with live *P. gingivalis *for 24 hours exhibit apoptosis, and we characterize this by M30 epitope detection, caspase-3 activity, DNA fragmentation and Annexin-V staining. Live bacteria strongly upregulated intrinsic and extrinsic apoptotic pathways. Pro-apoptotic molecules such as caspase-3, -8, -9, Bid and Bax were upregulated after 24 hours. The anti-apoptotic Bcl-2 was also upregulated, but this was not sufficient to ensure cell survival. The main *P. gingivalis *proteases arginine and lysine gingipains are necessary and sufficient to induce host cell apoptosis. Thus, live *P. gingivalis *can invoke gingival epithelial cell apoptosis in a time and dose dependent manner with significant apoptosis occurring between 12 and 24 hours of challenge via a gingipain-dependent mechanism.

**Conclusion:**

The present study provides evidence that live, but not heat-killed, *P. gingivalis *can induce apoptosis after 24 hours of challenge in primary human gingival epithelial cells. Either arginine or lysine gingipains are necessary and sufficient factors in *P. gingivalis *elicited apoptosis.

## Background

Chronic inflammatory periodontal disease is initiated by a bacterial biofilm called dental plaque that causes inflammation affecting the supporting structures of teeth, leading eventually to bone and tooth loss. *Porphyromonas gingivalis *is a Gram-negative anaerobe of dental plaque and a putative pathogen in chronic periodontitis [1]. The plaque bacteria possess numerous virulence factors including factors that aid intracellular invasion, intracellular persistence and host cell apoptosis [2].

Apoptosis or programmed cell death is triggered by two distinct signaling pathways; the intrinsic or stress-activated and the extrinsic or receptor-activated apoptotic pathway [3]. Both pathways activate their respective initiator caspases and converge to trigger executioner caspases 3, 6 and 7. The caspase cascade cleaves key cellular components responsible for the hallmarks of apoptosis such as chromatin condensation, pyknosis DNA fragmentation, cytoskeleton collapse, blebbing and formation of apoptotic bodies. Apoptosis is prevalent in the gingiva at sites of chronic bacteria-induced inflammation [4,5], particularly in the superficial cells of the junctional epithelium [5] and the fibroblasts and leucocytes of the connective tissue [4,5]. *In vitro *studies show that *P. gingivalis *can modulate apoptosis in the following cell types: fibroblasts [6,7], endothelial cells [8-11] and lymphocytes [12] and apoptosis has been proposed as a mechanism to explain the extensive tissue destruction in chronic periodontitis lesions.

It is not clear how *P. gingivalis *influences apoptosis in epithelial cells. In agreement with studies in fibroblasts, endothelial cells, cardiac myoblasts and lymphocytes, several authors [13,14] have shown induction of apoptosis in epithelial cells. In contrast, other laboratories [15-17] have shown inhibition of apoptosis by *P.gingivalis*. The reason for the discrepancies between these studies remains unknown, although variable challenge conditions were used. In this regard, the dose of bacteria and the duration of *P. gingivalis *challenge may be a critical parameter in determining whether induction or inhibition of apoptosis will occur. Thus, the aim of the current study was to characterize *P. gingivalis*-induced apoptosis of epithelial cells under various conditions, utilizing a wide array of apoptosis assays and gene expression profiling.

## Results

### HGECs challenged with live *P. gingivalis *show early signs of apoptosis in a time- and dose-dependent manner

**Figure 1:**
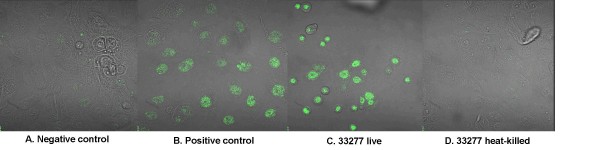
**M30 epitope immunohistochemistry was used to detect caspase-cleaved cytokeratin-18 which is detectable in early stages of apoptosis**. Images are fluorescent confocal staining at ×600 magnification. The negative control was unchallenged HGECs with only media added (A). The positive control was HGECs treated with camptothecin 4 μg/ml for 4 hours (B). HGECs challenged with live *P. gingivalis *33277 at MOI:100 for 24 hours show marked staining (C), while HGECs challenged with heat-killed bacteria under the same conditions show no detectable apoptosis (D). Challenging HGECs with an MOI:100 for 4 hours or MOI:10 for 4 and 24 hours showed no positive staining (no apoptosis) (data not shown).

HGECs were challenged with live or heat-killed *P. gingivalis *33277 at an MOI:10, MOI:100 and MOI:1000 for 4 and 24 hours and M30 epitope detection was performed with immunohistochemistry. M30 is an antibody that recognizes a specific caspase cleavage site within cytokeratin 18 that is not detectable in native cytokeratin 18 of vital cells. This occurs early in the apoptosis cascade, before Annexin-V reactivity or positive DNA nick labeling. Untreated cells were used as a negative control and cells treated with camptothecin 4 μg/ml for 4 hours, an apoptosis-inducing agent, were the positive control. Cells challenged with live or heat-killed bacteria at an MOI:10 showed no positive staining at any time point (data not shown). Cells challenged with live or heat-killed bacteria at an MOI:100 and MOI:1000 did not show any positive staining at 4 hours (data not shown). The epithelial cells appeared morphologically normal under all of the above conditions. However, challenge with live *P. gingivalis *at an MOI:100 for 24 hours increased the detachment of cells, while the remaining attached cells showed signs of blebbing, had pyknotic nuclei, and stained positive for M30 epitope, an early sign of apoptosis (Fig. 1C). In contrast, cells challenged with heat-killed *P. gingivalis *at an MOI:100 for 24 hours did not show any signs of apoptosis (Fig. 1D). Cells challenged with live *P. gingivalis *at an MOI:1000 for 24 hours completely detached from the plate, thus MOI:1000 was not used for subsequent experiments.

### Live but not heat-killed *P. gingivalis *induce caspase-3 activation in HGECs in a time-dependent manner

**Figure 2:**
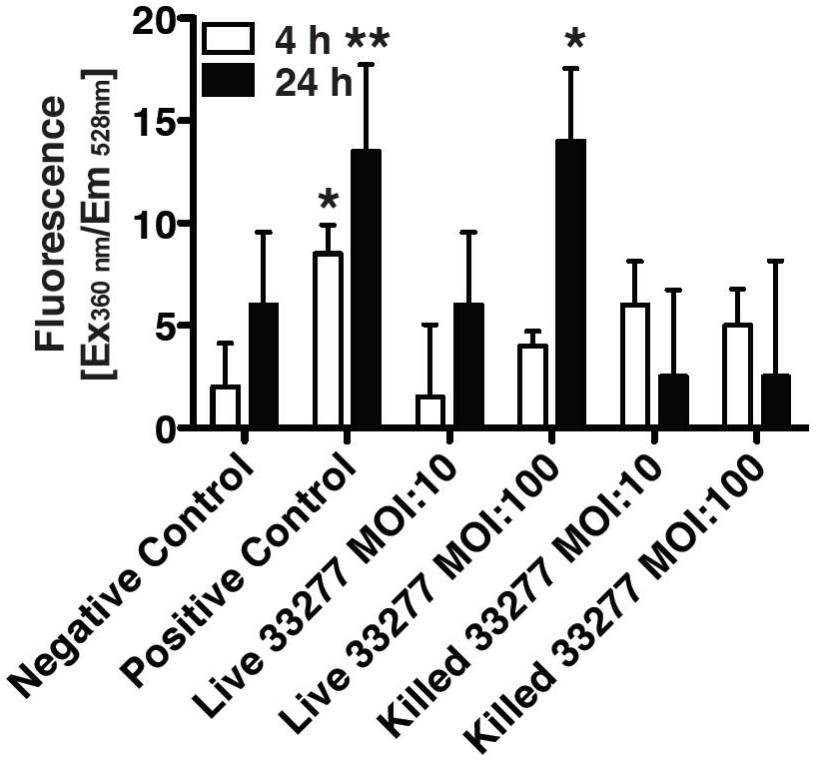
**FIENA was used to detect caspase-3 activation, a key molecule in initiation of apoptosis**. HGECs were challenged with live or heat-killed *P. gingivalis *33277 at MOI:10 and MOI:100 for 4 and 24 hours. Negative control was unchallenged HGECs. Positive control was HGECs challenged with camptothecin 4 μg/ml. Values represent the means ± SD of at least two experiments. Statistical comparisons are to the unchallenged negative control cells (* *P *< 0.05, ** *P *< 0.01).

HGECs were challenged with live or heat-killed *P. gingivalis *33277 at an MOI:100 for 4 and 24 hours and caspase-3 activity was measured fluorometrically. Caspase-3 is an executioner caspase involved in both the extrinsic and intrinsic pathway of apoptosis. Caspase-3 activation plays a key role in the initiation of cellular events during the early apoptotic process. Untreated cells were used as a negative control and cells treated with camptothecin were the positive control. There was no significant increase in caspase-3 activity after 4 hours challenge with live or heat-killed bacteria (Fig. 2). However, after 24 hours challenge with live *P. gingivalis*, caspase-3 activity increased more than 2-fold compared to the negative control. In contrast, 24 hours challenge with heat-killed *P. gingivalis *resulted in a caspase-3 activity level similar to the negative untreated control. These results are in accordance with our previous results, confirming that challenge with live, but not heat-killed, *P. gingivalis *at an MOI:100 for 24 hours can induce apoptosis in human gingival epithelial cells.

### HGECs challenged with live *P. gingivalis *undergo DNA fragmentation in a time- and dose-dependent manner

**Figure 3:**
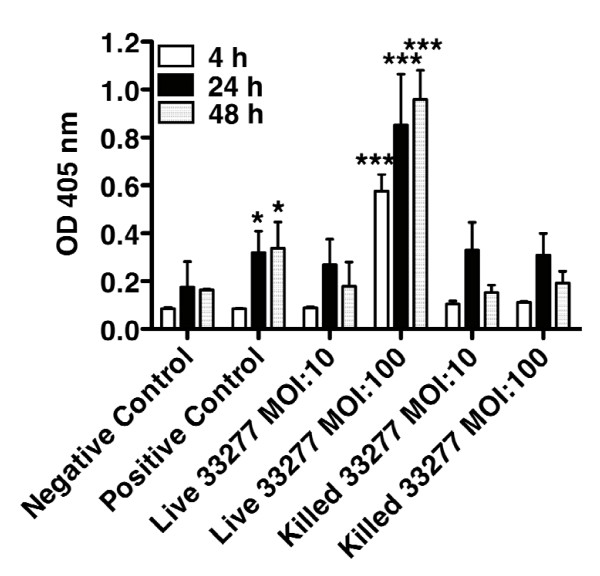
**Cell Death Detection ELISA was used to detect DNA fragmentation, a hallmark of apoptosis**. HGECs were challenged with live and heat-killed *P. gingivalis *33277 at MOI:10 and MOI:100 for 4, 24, and 48 hours. Negative control was unchallenged HGECs in media. Positive control was HGECs challenged with camptothecin 4 μg/ml. Values represent the means ± SD of at least two experiments. Statistical comparisons are to the unchallenged negative control cells (* *P *< 0.05, ** *P *< 0.01).

**Figure 4:**
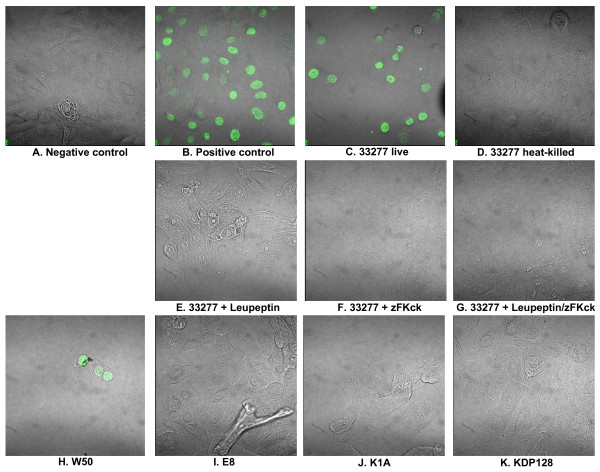
**TUNEL assay to detect DNA fragmentation by confocal microscopy**. Images are fluorescent confocal staining at ×600 magnification. Negative control was unchallenged HGECs (A). Positive control was HGECs treated with DNase 1000 U/ml (B). HGECs were challenged with live (C) and heat-killed (D) *P. gingivalis *33277 MOI:100 for 24 h. Challenge with MOI:100 for 4 h and MOI:10 for 4 and 24 h gave no staining (data not shown). Additional plates (E to G) show challenge with live *P. gingivalis *33277 at MOI:100 for 24 h that were pretreated with leupeptin, a selective Rgp inhibitor (E), zFKck, a selective Kgp inhibitor (F), or a cocktail of both inhibitors to inhibit total gingipain activity (G). Challenge with *P. gingivalis *W50 (H), the RgpA/RgpB mutant E8 (I), the Kgp mutant K1A (J) or the RgpA/RgpB/Kgp mutant KDP128 (K), at MOI:100 for 24 h are also shown.

HGECs were challenged with live or heat-killed *P. gingivalis *33277 at an MOI:10 and MOI:100 for 4, 24 and 48 hours and DNA fragmentation was detected by ELISA, as well as by TUNEL. Untreated cells were used as a negative control and cells treated with camptothecin or DNase 1000 U/ml were used as a positive control. Once the caspase cascade has been activated, the inhibitor of caspase-activated DNase (ICAD) is cleaved liberating this DNase and resulting in fragmentation of the chromosomal DNA. The Cell Death Detection ELISA can detect internucleosomal degradation of genomic DNA during apoptosis and provide relative quantification of histone-complexed DNA fragments (mono- and oligo-nucleosomes). There was no significant increase in DNA fragmentation after 4 hours challenge with live or heat-killed bacteria (Fig. 3). However, 24 hours challenge with live *P. gingivalis*, resulted in DNA fragmentation 3-fold higher than the negative control. On the other hand, 24 hours challenge with heat-killed *P. gingivalis *resulted in negligible increase in DNA fragmentation, suggesting that, although some apoptosis is evident after challenge with heat-killed bacteria, the effect is not statistically significant (Fig. 3). At 48 hours, DNA fragmentation was at similar levels as at 24 hours. These results were also confirmed by TUNEL. The TUNEL assay measures and quantifies apoptosis by labeling and detection of DNA strand breaks in individual cells by fluorescence microscopy. The assay uses an optimized terminal transferase (TdT) to label free 3'OH ends in genomic DNA. Cells challenged with live or heat-killed bacteria at an MOI:10 did not show any positive staining at any time point (data not shown). Cells challenged with live or heat-killed bacteria at an MOI:100 did not show any positive staining at 4 hours (data not shown). The epithelial cells appeared morphologically normal under all of the above conditions. However, the cells challenged with live *P. gingivalis *at an MOI:100 for 24 hours showed signs of blebbing and pyknotic nuclei and stained positive for TUNEL (Fig. 4C), confirming our previous observations using M30 epitope detection (Fig. 1C). In contrast cells challenged with heat-killed *P. gingivalis *at an MOI:100 for 24 hours did not show any signs of DNA fragmentation (Fig. 4D).

### *P. gingivalis*-induced apoptosis in HGECs is dependent on either Arg- or Lys- gingipains

**Figure 5:**
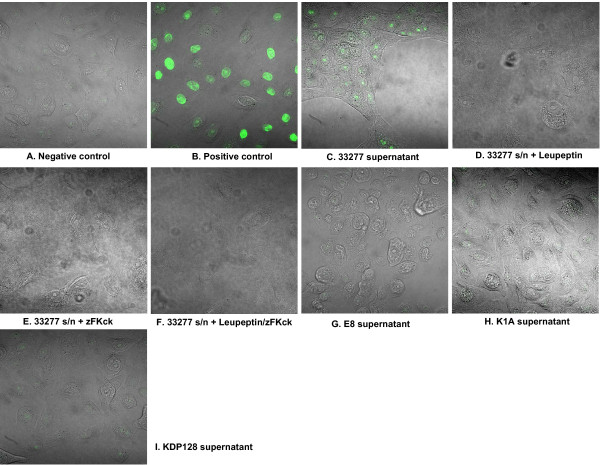
**TUNEL assay to detect DNA fragmentation by confocal microscopy**. Images are fluorescent confocal staining at ×600 magnification. Negative control was unchallenged HGECs at 24 h (A). Positive control was HGECs treated with DNase 1000 U/ml (B). HGECs were challenged with filtered supernatant of *P. gingivalis *33277 culture (C) for 24 h. Additional plates (D to F) show challenge with live *P. gingivalis *33277 supernatant pretreated with leupeptin, a selective Rgp inhibitor (D), zFKck, a selective Kgp inhibitor (E), or a cocktail of both inhibitors to inhibit total gingipain activity (F). Challenge for 24 hours with filtered culture supernatant derived from the RgpA/RgpB mutant E8 (G), the Kgp mutant K1A (H) or the RgpA/RgpB/Kgp mutant KDP128 (I), are also shown.

**Figure 6:**
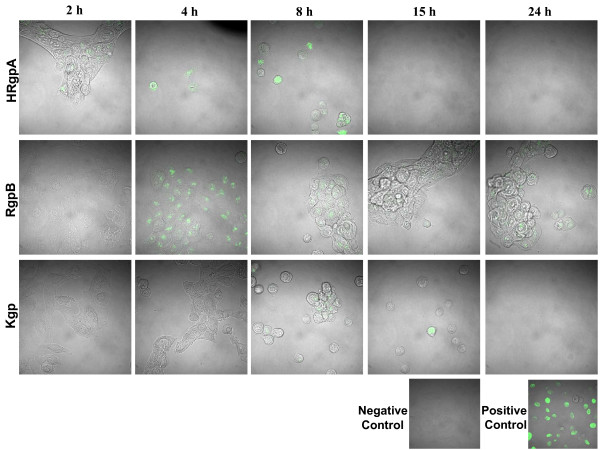
**TUNEL assay to detect DNA fragmentation by confocal microscopy**. Images are fluorescent confocal staining at ×600 magnification. Negative control was unchallenged HGECs at 24 h. Positive control was HGECs treated with DNase 1000 U/ml. HGECs were challenged with purified HRgpA (8 μg/ml), RgpB (5.2 μg/ml) and Kgp (3 μg/ml) (equivalent to 113 units of Rgp activity/ml or 12.4 units of Kgp activity/ml) for 2, 4, 8, 15 and 24 h.

**Figure 7:**
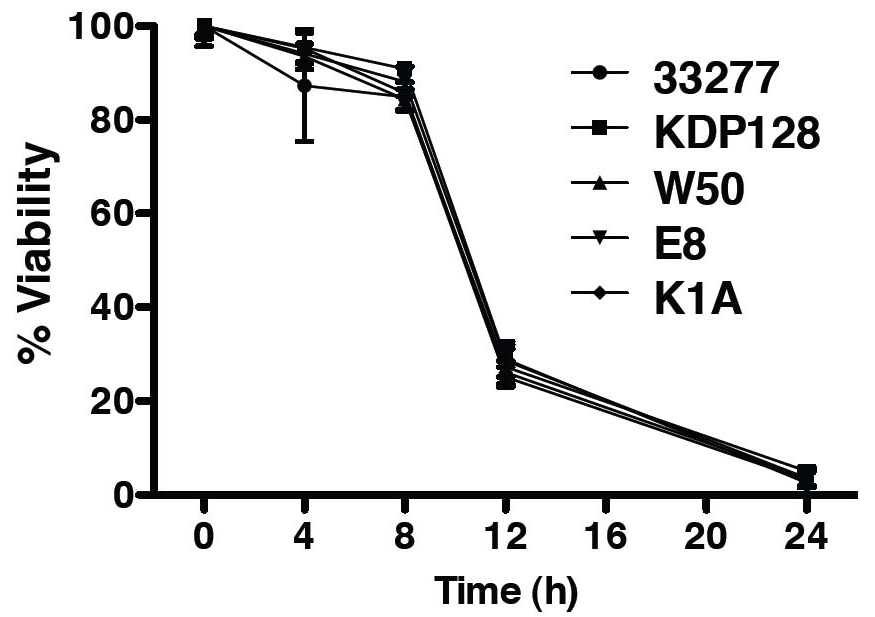
**Bacterial viability was determined following epithelial cell challenges**. From each challenge assay reported in Fig. 4, supernatant containing bacteria was removed at 4, 8, 12, and 24 hours, plated in blood agar plates and colony forming units were counted. *P. gingivalis *strains cultured were 33277, KDP128, W50, E8 and K1A. For all strains > 80% viability persisted until 8 h, where upon viability decreased to approximately 30% at 12 h and 1–2% at 24 h. Values represent the means ± SD of at least two experiments.

*P. gingivalis*-induced apoptosis has been shown previously to depend on gingipain activity in fibroblasts and endothelial cells [7,8,10,11]. Gingipains are cysteine proteases produced by *P.gingivalis *that cleave after an arginine (Arg) or a lysine (Lys) residue. To elucidate the role of gingipains in our *P. gingivalis*-induced apoptosis model, HGECs were challenged with whole live bacteria (Fig. 4) as well as filtered bacterial supernatant (Fig. 5) of the following strains: wild-type *P. gingivalis *33277; wild-type W50; the Arg-gingipain (RgpA/RgpB) double mutant E8; the Lys-gingipain (Kgp) mutant K1A; or the Arg-Lys-gingipain (RgpA/RgpB/Kgp) triple mutant KDP128. All strains were utilized live at an MOI:100 and the filtered supernatants at a 10× dilution. DNA fragmentation was assessed by TUNEL after 24 hours. HGECs were also challenged with live wild-type *P. gingivalis *33277 or its filtered supernatant previously incubated with leupeptin, a specific Rgp inhibitor, zFKck, a specific Kgp inhibitor, or a cocktail of both gingipain inhibitors. Untreated cells were used as a negative control and cells treated with DNase 1000 U/ml were used as a positive control. Increased detachment of cells was observed upon challenge with 33277 and W50 detached from the plate, while the remaining cells showed signs of blebbing and pyknotic nuclei and stained positive for TUNEL (Fig. 4C, H), confirming our previous observations on live wild-type *P. gingivalis*. When any of the gingipain deficient mutants was used for the live challenge, DNA fragmentation was not evident (Fig. 4I, J, K, Fig. 5G, H, I), suggesting that the presence of either Arg- and Lys- gingipains is necessary for apoptosis and that depletion of any one of them completely abolishes *P. gingivalis*-induced apoptosis in HGECs (Fig. 6). Furthermore, cell detachment was still observed to a lesser extent with both E8 and K1A, suggesting that apoptosis is independent of cell detachment (Fig. 4I, J, K). The difference between the strains is unlikely to be due to differences in bacterial viability, since the viability over time in culture was similar for all strains examined (Fig. 7). The role of gingipains in HGEC apoptosis was also confirmed by using specific gingipain inhibitors (Fig. 4E, F, G). Furthermore, apoptosis was still observed when HGECs were challenged with filtered supernatant of *P. gingivalis *33277 culture (Fig. 5C), but not when the challenge was performed with supernatant pre-incubated with gingipain inhibitors (Fig. 5D, E, F) or supernatant derived from the gingipain-deficient mutants (Fig. 5G, H, I). These results suggest that apoptosis is not dependent on bacterial invasion and although invasion might influence the apoptotic process our data reaffirm that gingipains are sufficient to invoke this process.

### Purified gingipains can induce detachment and apoptosis in HGECs

Our previous experiments with live bacteria and bacterial culture supernatant suggest that either Arg- or Lys-gingipains are necessary for apoptosis in HGECs. In order to determine if specific purified gingipains are also sufficient to induce apoptosis, HGECs were challenged with purified HRgpA, RgpB and Kgp for 2, 4, 8, 15 and 24 hours and DNA fragmentation was assessed by TUNEL (Fig. 6). All three gingipains were able to induce cell detachment and apoptosis, although at different time points. For HRgpA, signs of apoptosis were already evident at 2 hours post-challenge, while for RgpB and Kgp, TUNEL positive cells appeared at 4 and 8 hours respectively. For all three gingipains, the percentage of apoptotic and detached HGECs increased progressively over time. By 24 hours, HGECs challenged with HRgpA and Kgp had completely detached from the plates, while some clumped cells still remained on the plates challenged with RgpB (Fig. 6).

### Different WT *P. gingivalis *strains induce apoptosis with similar kinetics

**Figure 8:**
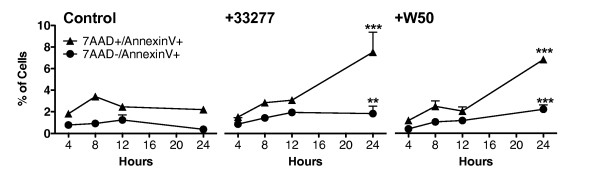
**Flow cytometry for Annexin-V staining to detect PS externalization, an early apoptotic event**. HGECs were challenged with live WT *P. gingivalis *33277 and W50 at MOI:100 for 4, 8, 12, and 24 hours. The percent of apoptotic cells (7AAD+/AnnexinV+ and 7AAD-/AnnexinV+) is shown for unchallenged HGECs (control), and HGECs challenged with each of the WT strains (+33277, +W50). Values represent the means ± SD of at least two experiments. Statistical comparisons are between challenged and control cells at the same time points ** *P *< 0.01, *** *P *< 0.001.

HGECs were challenged with live *P. gingivalis *33277 or W50 at an MOI:100 for 4, 8, 12 and 24 hours and phosphatidylserine (PS) externalization was measured by Annexin-V staining. Untreated cells were used as a negative control. A slow gradual increase in both Annexin-V single and Annexin-V/7-AAD double positive cells was noted for HGECs challenged with both strains compared to the unchallenged control over 12 hours (Fig. 8). The percentage of apoptotic cells was 4–5 fold higher than the unchallenged control 24 hours after challenge with either WT strain. The results of this kinetic study confirm our previous observations that apoptosis occurs late upon *P. gingivalis *challenge. Furthermore, the similarity in the kinetics of the response between the two strains suggests that the observed apoptosis is a characteristic of *P. gingivalis *and not an attribute of a single strain.

### *P. gingivalis *challenge of HGECs results in upregulation of genes related to apoptosis

**Figure 9:**
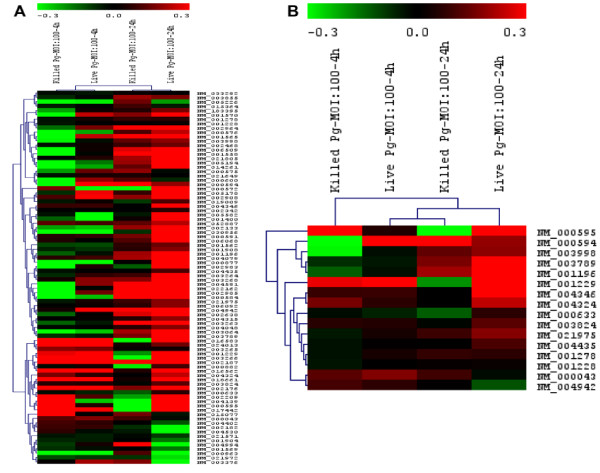
**Focused qPCR-Array consisting of 86 genes relevant to inflammation and apoptosis**. HGECs were challenged with live or heat-killed *P. gingivalis *33277 at MOI:100 for 4 and 24 hours. Negative control was unchallenged HGECs in media. The mRNA fold change between each sample and the negative control was calculated based on the ΔΔCt method and Log10 fold-increase was used to generate the heatmap using MeV v4.1 release software and hierarchical clustering with Pearson correlation. (A) represents a heatmap of the 86 genes and (B) represents specific apoptotic markers with color coding: Magenta (up-regulated genes) to Green (down-regulated genes). The apoptotic markers in (B) and the fold differences are shown in Table 1.

HGECs were challenged with live or heat-killed *P. gingivalis *33277 at an MOI:100 for 4 and 24 hours and qPCR was performed on a focused panel of 86 apoptosis-related genes (Fig. 9). Live bacteria strongly upregulated apoptosis related pathways, as indicated by caspase cascade activation and apoptotic signaling activation in response to DNA damage. More specifically, the pro-apoptotic molecules caspase-3, -8, -9, Bid and Bax were upregulated at 4 and strongly upregulated at 24 hours, while the anti-apoptotic Bcl-2 was also upregulated at 24 hours. Both the intrinsic and extrinsic pathways appear to be involved, as indicated by the activation of mitochondrial apoptosis signaling, as well as the Fas signaling pathway, TNFR and IL-1R signaling pathways (TNF, TRADD, FADD, IL-1b, IL-1R1, IRAK-2). The effect of heat-killed bacteria was less pronounced, indicating that higher doses or longer challenge times would be necessary to induce apoptosis.

## Discussion

We demonstrate that primary HGECs challenged with live *P. gingivalis *for 24 hours exhibit apoptosis, evidenced by M30 epitope detection, caspase-3 activity, DNA fragmentation and Annexin-V staining. Apoptosis was dose and time dependent and live bacteria strongly upregulated apoptotic intrinsic and extrinsic pathways, including the pro-apoptotic molecules caspase-3, -8, -9, Bid and Bax. Arginine and lysine gingipains are clearly essential factors in apoptosis and depletion of either inhibits apoptosis.

In the present study, live *P. gingivalis *induced considerable apoptosis in human gingival epithelial cells between 12 and 24 hours at MOI:100, as evidenced by M30 epitope detection (Fig. 1), increased caspase-3 activity (Fig. 2), DNA fragmentation (Fig. 3, Fig. 4) and Annexin-V staining (Fig. 8). These results agree with previous reports on fibroblasts [7,18], endothelial cells [9] and lymphocytes [12]. In contrast, heat-killed *Porphyromonas gingivalis *did not induce apoptosis.

Apoptosis is a complex process regulated by multiple pathways such that no single molecule gives sufficient information on the dynamics of apoptosis. After an apoptotic stimulus, a subset of pro-apoptotic molecules is upregulated and others such as Bcl-2, an anti-apoptotic molecule, downregulated, with cellular fate depending on the fine tuning of all pathways involved. We used a focused array of 86 apoptosis-related genes to elucidate the apoptotic process (Fig. 9). Live *P. gingivalis *strongly upregulated apoptosis pathways: evidenced by caspase cascade activation and apoptotic signaling in response to DNA damage. Both the intrinsic and extrinsic pathways appear to be involved in this process: evidenced by activation of mitochondrial apoptosis signaling, as well as Fas signaling, TNFR signaling and IL-1R signaling pathway (Table 1). In terms of individual molecules, the pro-apoptotic caspase-3, -8, -9, Bid, Bax, TNF, TRADD, FADD, IL-1b, IL-1R1, IRAK-2 were upregulated after 24 hours. On the other hand, the anti-apoptotic Bcl-2 was also upregulated, but this did not appear to be sufficient to ensure cell survival, as indicated by the apoptosis assays (Fig. 1, Fig. 2, Fig. 3, Fig. 4, Fig. 8). The upregulation of Bcl-2 is in agreement with Nakhjiri *et al *[16], underlining the fact that single molecule and single time point assessments alone can be misleading.

**Table 1:** Apoptotic markers included in the qPCR-Array shown in Fig. 1.

Genes	Killed *Pg *MOI:100 4 h	Killed *Pg *MOI:100 24 h	Live *Pg *MOI:100 4 h	Live *Pg *MOI:100 24 h
**LTA**	4.7 ± 3.4**	0.4 ± 0.1***	1.1 ± 0.8*	3.8 ± 1.2**
**TNF**	0.4 ± 0.01	2.0 ± 0.01**	2.1 ± 0.2***	1.6 ± 0.1***
**NFKB1**	0.5 ± 0.01	1.4 ± 0.03	0.9 ± 0.1*	1.5 ± 0.05*
**TRADD**	0.8 ± 0.01	1.5 ± 0.3**	0.9 ± 0.2	3.4 ± 0.1***
**BID**	0.7 ± 0.02	1.6 ± 0.1***	0.9 ± 0.1*	3.1 ± 0.08***
**CASP9**	1.9 ± 0.7**	0.6 ± 0.2*	2.4 ± 1.1**	2.2 ± 0.2**
**CASP3**	1.2 ± 0.02*	1.0 ± 0.01	1.2 ± 0.1*	2.2 ± 0.4***
**BAX**	1.5 ± 0.5*	1.0 ± 0.08	1.2 ± 0.01	1.7 ± 0.8**
**BCL2**	0.9 ± 0.02**	0.7 ± 0.02**	0.9 ± 0.1**	1.2 ± 0.7*
**FADD**	1.2 ± 0.01	1.0 ± 0.01	1.2 ± 0.1*	1.3 ± 0.05**
**RELA**	0.9 ± 0.03**	1.2 ± 0.05**	1.1 ± 0.08*	1.5 ± 0.1***
**ENDO-G**	0.9 ± 0.01	1.0 ± 0.01	1.0 ± 0.1	1.3 ± 0.1**
**CHUK**	0.9 ± 0.06*	1.2 ± 0.08**	1.1 ± 0.1*	1.2 ± 0.3**
**CASP8**	0.9 ± 0.01**	1.0 ± 0.07	1.0 ± 0.1	1.1 ± 0.1**
**FASLG**	1.3 ± 0.02	1.3 ± 0.02**	1.5 ± 0.1**	0.9 ± 0.2**
**DFFB**	1.3 ± 0.03**	1.0 ± 0.1	1.2 ± 0.2*	0.8 ± 0.01

HGECs were challenged with live or heat-killed *P. gingivalis *33277 at MOI:100 for 4 and 24 hours. Negative control was unchallenged HGECs in media. The data shown represent log-fold differences in gene expression (means ± SD) between the respective sample and the negative control. A value of 1 indicates no change, less than one indicates down-regulation and greater than one, up-regulation (*P < 0.05 ** *P *< 0.01, *** *P *< 0.001)

It has been suggested that apoptosis due to *P. gingivalis *challenge of human cells involves the gingipains [7,8,10,11,14]. Gingipains are cysteine proteases produced by *P. gingivalis *that are either secreted or membrane bound and arginine or lysine specific. In the present study, the mechanism used by *P. gingivalis *to induce apoptosis in gingival epithelial cells was shown to be dependent upon both Arg- and Lys- gingipains (Fig. 4). Gingipain deficient *P. gingivalis *mutants did not cause apoptosis as evidenced by a lack of DNA fragmentation indicating that gingipains are necessary for apoptosis to occur and that their depletion abolishes *P. gingivalis*' ability to induce apoptosis in HGECs. This suggests a step-wise enzymatic action of these gingipains on substrates such that action of one alone is not sufficient. Similarly, inhibition of apoptosis was also observed when the wild-type *P. gingivalis *was pre-treated with specific gingipain inhibitors, providing evidence that the observed lack of apoptosis is due to the lack of gingipains and not other potential differences between the wild-type strains and the mutants.

Furthermore, filtered cell-free supernatant derived from wild-type *P. gingivalis *culture, as well as purified gingipains, retained the ability to induce apoptosis in HGECs (Fig. 5, Fig. 6), providing evidence that the gingipains are sufficient for the induction of apoptosis and that the presence of whole cells is not necessary for this process. This suggests that apoptosis is not dependent on bacterial invasion and although invasion might influence the apoptotic process our data reaffirm that gingipains are sufficient to invoke this process. The ability of the bacterial culture supernatant to induce apoptosis was lost when it was pre-incubated with specific gingipain inhibitors, while bacterial culture supernatant derived from gingipain-deficient mutants did not result in apoptosis (Fig. 5). These results are in agreement with previous studies in endothelial cells [10,11]. The mechanism of action of gingipains has been shown to be both caspase-dependent and caspase-independent [11] and *in vitro *evidence suggests that gingipains may activate caspase-3 by cleaving procaspase-3 [7].

In addition to variable bacterial strain virulence and variable host resistance, local factors, such as MOI or length of exposure, could vary across different areas of the lesion and inter-laboratory differences in apoptosis studies may reflect these variables. Thus, results from different laboratories and studies may supplement rather than conflict each other in elucidating the actions of *P. gingivalis *on host epithelial cells. In areas where the bacteria to epithelial cells ratio is low or the exposure time is short, bacterial invasion [19,20] may result in cell survival [15-17], contributing to the chronicity of the periodontal lesion. On the other hand, in areas with high bacteria to epithelial cell ratio or longer exposure time, the bacterial insult may result in apoptosis [7,9], contributing to extensive tissue destruction. Further translational studies are needed to determine which scenarios predominate in the pathogenesis of periodontitis.

## Conclusion

The present study provides evidence that live, but not heat-killed, *P. gingivalis *can induce apoptosis after 24 hours of challenge in primary human gingival epithelial cells. Either arginine or lysine gingipains are necessary and sufficient factors in *P. gingivalis *elicited apoptosis.

## Methods

### Cell isolation and culture

Gingival tissue biopsies were obtained with informed consent from periodontally healthy patients undergoing crown lengthening procedures at the University of Louisville School of Dentistry Graduate Periodontics Clinic, according to an IRB approval. The gingiva was treated with 0.025% trypsin and 0.01% EDTA overnight at 4°C and human gingival epithelial cells (HGECs) were isolated as previously described [21]. The HGECs were seeded in 60-mm plastic tissue culture plates coated with type-I collagen (BD Biocoat, Franklin Lakes, NJ, USA) and incubated in 5% CO_2 _at 37°C using K-SFM medium (Invitrogen, Carlsbad, CA, USA) containing 10 μg/ml of insulin, 5 μg/ml of transferrin, 10 μM of 2-mercaptoethanol, 10 μM of 2-aminoethanol, 10 mM of sodium selenite, 50 μg/ml of bovine pituitary extract, 100 units/ml of penicillin/streptomycin and 50 ng/ml of fungizone (complete medium). When the cells reached sub-confluence, they were harvested and sub-cultured as previously described [22].

### Bacterial strains and conditions

*P. gingivalis *ATCC 33277 was purchased from the ATCC (Manassas, VA, USA) and the derivative KDP128, an RgpA/RgpB/Kgp triple mutant [23], was kindly provided by Dr. K. Nakayama (Nagasaki University Graduate School of Biomedical Sciences). *P. gingivalis *W50 (ATCC 53978), and the derivative mutants E8, an RgpA/RgpB double mutant, and K1A, a Kgp mutant [24], were kindly provided by Dr. M. Curtis (Barts and The London, Queen Mary's School of Medicine and Dentistry). All *P. gingivalis *strains at low passage were grown in GAM media (Nissui Pharmaceutical, Tokyo, Japan) under anaerobic conditions (85% N_2_, 10% CO_2 _and 10% H_2_; Coy Laboratory) for 2 days. After cultivation, the bacteria were harvested by centrifugation, washed in PBS (pH 7.4) and used immediately for the live cell challenge or heat-inactivated for 1 h at 60°C. For the bacterial culture supernatant assays, the supernatant was filtered sterilized using a 0.22 μm pore PVDF membrane (Millipore, USA). The Rgp and Kgp activity of each strain was determined using the enzymatic substrate hydrolysis of N-α-benzoyl-DL-arginine-p-nitroanilide (BAPNA) (Sigma), for Rgp activity, or acetyl-lysine-p-nitroanilide (ALNA) (Bachem), for Kgp activity. The Rgp and Kgp activity were negligible for the heat-killed bacteria.

### Purified gingipains and gingipain inhibitors

Purified HRgpA, RgpB and Kgp were isolated as previously described [25-27]. The purified gingipains were used at a final concentration of 8 μg/ml for HRgpA, 5.2 μg/ml for RgpB and 3 μg/ml for Kgp (all equivalent to 113 units of Rgp activity/ml or 12.4 units of Kgp activity/ml) in the presence of 5 mM L-cysteine [10]. For the gingipain inhibition assays, live *P. gingivalis *33277 or its culture supernatant was incubated with gingipain inhibitors for 15 min at 37°C, just prior to the HGEC challenge. zFKck, a specific Kgp inhibitor [28], was used at a final concentration of 10 μM. Leupeptin (Sigma), a specific Rgp inhibitor, was used at a final concentration of 100 μM. The final concentrations used were the maximum inhibitory doses that retained specificity, as determined by a dose-response using the enzymatic substrate hydrolysis of N-α-benzoyl-DL-arginine-p-nitroanilide (BAPNA) (Sigma), for Rgp activity, or acetyl-lysine-p-nitroanilide (ALNA) (Bachem), for Kgp activity.

### Bacterial challenge

HGEC cultures at the fourth passage were harvested and seeded at a density of 0.5 × 10^5 ^cells/well in a 6-well culture plate coated with type-I collagen or in a 35-mm collagen-coated glass bottom culture dishes (Mat-tek Corp., Ashland, MA, USA), and maintained in 2 ml of complete medium. When they reached confluence (approximately 10^6 ^cells/well), the cells were washed twice with fresh media and were challenged with live or heat-inactivated bacteria in antibiotic-free medium at MOI:10 (10^7 ^bacteria/well) and MOI:100 (10^8 ^bacteria/well) at 37°C in 5% CO_2 _for 4 or 24 hours. For each experiment the final concentration of the suspension was determined by measurement of A_600 _and appropriate dilutions were made to achieve the desired MOI. The bacterial number was confirmed by viable counting of colony forming units (cfu) on blood agar plates incubated at anaerobically at 37°C.

### M30 epitope detection

The M30 epitope released by caspase-cleaved cytokeratin-18 was detected using a commercially available kit (CytoDEATH Fluorescein kit, Roche Applied Science, Indianapolis, IN, USA), according to the manufacturer's instructions. Briefly, the cells were washed three times with PBS, fixed with ice-cold pure methanol for 30 minutes at -20°C and then incubated with the M30 antibody for 60 minutes at room temperature. After three washes, the cells were observed on a confocal microscope (Olympus Fluoview 500, Center Valley, PA, USA).

### Caspase-3 activity assay

Caspase-3 activity was determined by FIENA (Fluorometric Immunosorbent Enzyme Assay) using a commercially available kit (Roche Applied Science, Indianapolis, IN, USA) according to the manufacturer's instructions. Briefly, after centrifugation of the 6-well plates, the supernatant was discarded and the cells were incubated in lysis buffer for one minute on ice. After centrifugation, the cell lysate was collected, added into the anti-caspase 3 coated microplate, and incubated for 60 minutes at 37°C. After washing, the caspase substrate was added and incubated for 24 h at 37°C. The fluorescence was measured at 360/528 nm.

### DNA fragmentation assay

Histone associated DNA fragments were detected using a commercially available kit (Cell Death Detection ELISA, Roche Applied Science, Indianapolis, IN, USA), according to the manufacturer's instructions. Briefly, after centrifugation of the 6-well plates, the supernatant was discarded and the cells were incubated in lysis buffer for 30 minutes at room temperature. After centrifugation, the cell lysate was collected and added into the streptavidin-coated microplate. Incubation with the monoclonal antibodies, anti-histone (biotin-labeled) and anti-DNA (peroxidase-conjugated), was followed by washing and incubation with peroxidase substrate. The absorbance was measured at 405 nm.

### TUNEL assay

Direct TUNEL (Terminal deoxynucleotidyl Transferase Fluorescein-dUTP Nick End Labeling) assay was performed using a commercially available kit (Roche Applied Science, Indianapolis, IN, USA), according to the manufacturer's instructions. Briefly, the cells were washed three times with PBS, fixed with 4% paraformaldehyde (pH 7.4) for 30 minutes at room temperature, washed twice, and then permeabilized with 0.1% Triton X (Sigma-Aldrich, St. Louis, MO, USA). After two washes, the cells were incubated with the TUNEL reaction mixture for 60 minutes at 37°C and then washed three times before analysis by confocal microscope (Olympus Fluoview 500, Center Valley, PA, USA).

### Annexin-V staining

Analysis of apoptosis was performed by flow cytometry using Alexa Fluor 488 Annexin-V (Molecular Probes, Invitrogen, USA). 7-AAD (eBioscience, San Diego, CA, USA) was used for the discrimination of dead cells. Briefly, the cells were dissociated with 0.025% trypsin and 0.01% EDTA, washed two times with PBS and incubated in 100 μl annexin-binding buffer containing 5 μl Alexa Fluor 488 Annexin-V for 15 minutes at room temperature. After washing in PBS, the samples were resuspended in 300 μl of annexin-binding buffer containing 5 μl 7-AAD and analyzed by flow cytometry using a FACSCalibur System (BD Biosciences, San Jose, CA, USA).

### Quantitative PCR Array

A focused panel of 86 apoptosis-related genes (qPCR-Array) was customized by SuperArray (Bioscience Corporation, Frederick, MD, USA) on a 96 well format including endogenous controls. The qPCR-Array was optimized for template and PCR conditions according to the manufacturer's recommendations. The total RNA was isolated and purified as described previously [29] and first strand cDNA was synthesized using the High Capacity cDNA Reverse Transcription Kit (Applied Biosystems, CA) according to the manufacturer's instructions. The real time PCR reaction cocktail was prepared by mixing 1125 μl of 2× SuperArray PCR master mix (RT^2 ^Real-Time™ SYBR Green/ROX (Cat. No. PA 012), 2 μg of cDNA, and 1127 μl of ddH_2_O. The final volume was adjusted to 2450 μl and 25 μl of the cocktail was loaded onto each well. 10 fold serial dilutions of experimental cocktail were used for β-actin gene to check the linearity and consistent amplification across the panels. The plate was loaded on to ABI 7500 Real Time PCR machine (Applied Biosystems, Foster City, CA, USA) and the reaction was carried out using relative quantification method with the following conditions: 1 cycle at 95°C for 10 minutes followed by 40 cycles of 15 seconds at 95°C and 1 minute at 60°C. The dissociation curve was drawn up after completing the relative quantification method which ensures specific amplification. The PCR-Array was duplicated for each sample and fold differences were calculated according to the ΔΔC_t _method using GAPDH as the endogenous control.

### Statistical analysis

All data are expressed as the mean ± SD. Statistical analyses were performed by one-way analysis of variance (ANOVA) using the InStat program (GraphPad, San Diego, CA, USA) with Bonferroni correction. Statistical differences were considered significant at the p < 0.05 level.

## Competing interests

The authors declare that they have no competing interests.

## Authors' contributions

DFK, JCG and PGS designed the study and drafted the manuscript. PGS carried out majority of the experiments. JCG carried out the apoptosis assays. MRB designed the PCR array experiments and helped in drafting the manuscript. CAG carried out the flow cytometry experiments. JP provided critical comments to improve the manuscript. All authors were involved in analyzing all the data, read and approved the final manuscript.
